# Characterization of key volatile compounds in meat-based broths using HS-SPME-Arrow-GC/MS and their relationship to sensory perception

**DOI:** 10.1016/j.fochx.2026.103601

**Published:** 2026-01-27

**Authors:** Jin-Kyung Nam, Mi-Ran Kim, Jeong Eun Hyeon, Hae Won Jang

**Affiliations:** a Department of Food Science and Biotechnology, Sungshin Women's University, Seoul 01133, Republic of Korea; b Department of Food Science and Nutrition, The Catholic University of Korea, Bucheon-si 14662, Republic of Korea

**Keywords:** Meat-based broth, Volatile compounds, Method optimization, Key volatile compound, Sensory evaluation

## Abstract

Meat-based broths are increasingly popular with consumers; however, their volatile profiles remain underexplored. This study uses headspace solid-phase microextraction-Arrow combined with gas chromatography/mass spectrometry to identify and quantify volatile compounds in commercial meat-based broths. The analytical method was optimized using response surface methodology and validated through calibration curves, limits of detection and quantification, and recovery analyses to ensure sensitivity and accuracy. Eighteen volatile compounds were identified, with aldehydes being the most abundant. Several compounds, including aldehydes contributing fatty characteristics, were identified in meat-based broths as well as beef flavoring ingredients (extract and powder), whereas pyrazines and methional were found only in beef flavoring ingredients. In sensory evaluations, samples with strong meaty odors and rich umami tastes received high ratings, whereas weak odors and flavors or low saltiness yielded poor consumer acceptance. By identifying key volatile compounds in meat-based broths, this study provides insight for developing Korean-style home meal replacement products.

## Highlights

• The first systematic volatile profiling of commercial meat-based broths is reported.
• Integrated chemical and sensory analyses reveal key flavor contributors.
• Key aldehydes (decanal, heptanal, hexanal, nonanal, and octanal) impart fatty notes.
• Strong correlations are observed between key aldehydes and consumer liking.

## Graphical abstract

**Figure:**
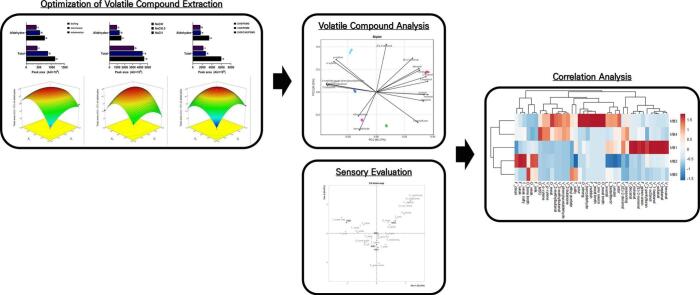

## 1 Introduction

The demand for pre-prepared meals intended for home consumption, such as ready-to-eat meals and home meal replacement (HMR) products, has increased steadily in recent decades, partly due to the growing number of single-person households. In the Korean market, recent developments in the HMR sector have emphasized soup- and stew-based products, particularly those that include meat or meat-derived components (Ahn et al., 2024). Bone broth has emerged as a fundamental base of these products, as it offers both rich flavor and important nutrients, including several amino acids and minerals that support physiological functions in humans (Zhang et al., 2017). For example, certain types of bone broth and soup have been reported to exhibit antioxidant properties, promote bone growth, and prevent osteoporosis (Meng et al., 2022).

Given the importance of flavor in shaping consumer preference and purchasing behavior, understanding the volatile profiles of HMR products is crucial. However, although over 1000 volatile compounds have been identified in different meats and meat-derived products (Ahamed et al., 2023), investigations into the volatile profiles of meat-based broths remain scarce. Most studies on meat-based broths have focused on characterizing non-volatile compounds, such as free amino acids and 5′-nucleotides (Wang et al., 2020; Yue et al., 2024). Meanwhile, little research has been conducted on the quantification of volatile compounds in meat-based broths and the identification of key contributors to their volatile profiles. Moreover, previous studies on bone broths have primarily focused on laboratory-prepared samples (Meng et al., 2022; Zhang et al., 2017), with limited attention given to the analysis of volatile compounds in commercial products.

The accurate identification and quantification of volatile compounds in bone broths is essential for understanding and enhancing the volatile profiles of HMR products. Headspace solid-phase microextraction-Arrow (HS-SPME-Arrow) combined with gas chromatography/mass spectrometry (GC/MS) is a reliable and sensitive technique for characterizing volatile compounds in foods (Lee et al., 2019). Headspace extraction is a solvent-free method with high sensitivity and selectivity (Nam et al., 2024), while solid-phase microextraction (SPME) offers increased speed, automation, and reproducibility (Lee et al., 2025). SPME-Arrow is a more recent advancement, integrating the benefits of SPME and stir bar sorptive extraction. Owing to its larger sorbent volume and improved fiber stability, SPME-Arrow significantly enhances the limits of detection (LODs) and GC/MS peak intensities, making it an effective tool for analyzing volatile compounds with high sensitivity and reproducibility (Lee et al., 2019; Nam et al., 2024).

In this study, HS-SPME-Arrow-GC/MS was used to extract, identify, and quantify volatile compounds in Korean-style meat-based broths and beef flavoring ingredients, with the aim of systematically characterizing the volatile compounds in meat-based broths, identifying key compounds, and understanding the volatile profiles of commercially available HMR products. The development and validation of analytical methods are crucial for advancing scientific understanding. Therefore, comprehensive method validation was performed using calibration curves, LODs, limits of quantification (LOQs), and recovery assessments. Instrumental analysis provides objective data on volatile profiles, but it does not reflect human sensory perception. To address this limitation, sensory evaluation was employed to obtain a more comprehensive understanding of how volatile profiles relate to human experiences of food aroma (Jung et al., 2025). This study provides the first characterization of key volatile compounds in Korean-style meat-based broths through instrumental analysis and sensory evaluation. The results offer insight into how the volatile compounds in meat-based broths and beef flavoring ingredients contribute to the overall volatile profiles of meat-based products.

## 2 Materials and methods

### 2.1 Sample preparation

Five commercial meat-based broths were obtained from a local supermarket in Seoul, Korea. The stated ingredients of each broth are listed in Table S1. Prior to volatile analysis, the broth samples were heated while sealed in their original packaging by submerging them in boiling water at 100 °C. After 2 min, the containers were turned over and heated for an additional 2 min. Beef extract and beef powder were provided by Seoul Flavor & Fragrance Co. (Seoul, Korea) and analyzed without modification. For sensory evaluation, the broth samples were filtered through an 80-mesh sieve to remove solid particles, yielding clear broth that was subsequently divided into 150 g portions and vacuum-sealed in sous vide bags (15 cm × 20 cm; Hooaco, Goyang, Korea). The sealed bags were heated in boiling water at 100 °C for 2 min, turned over, and heated for an additional 2 min. Following heating, the samples were transferred to white paper containers (Deakyung, Hwaseong, Korea) in 50 g portions. Sensory evaluation was performed with samples maintained at 60 °C.

### 2.2 Chemicals and reagents

All chemical standards for the detected volatile compounds and all internal standards (phenyl acetate, 1-hexyl alcohol-*d*_13_, toluene‑*d*_8_, 3-octanone, and 2,4,6-trimethylpyridine) were of analytical grade and obtained from Sigma-Aldrich (St. Louis, MO, USA). HPLC-grade water and methanol were purchased from Fisher Scientific (PA, USA).

### 2.3 Optimization of volatile compound extraction

To determine the optimal sample processing conditions, extraction experiments were conducted with different sample pretreatment (heating) methods, volumes of aqueous saturated NaCl solution, and SPME-Arrow fiber types. Specifically, pretreatment was performed by techniques such as submersion, microwave heating, and boiling; samples were prepared with 0, 0.5, and 1 mL of saturated NaCl solution; and extraction was performed with three types SPME-Arrow fiber, namely, DVB/PDMS, CAR/PDMS, and DVB/CAR/PDMS (where DVB = divinylbenzene, CAR = carbon, and PDMS = polydimethylsiloxane). All SPME-Arrow fibers had a phase length of 1.1 mm × 20 mm and phase thickness of 120 μm. The experimental conditions were optimized based on the GC/MS peak areas.

Next, to determine the optimal SPME-Arrow extraction conditions, the equilibration time (10, 20, and 30 min), extraction time (20, 30, and 40 min), and extraction temperature (30, 40, and 50 °C) were optimized using a Box–Behnken design (BBD) combined with response surface methodology (RSM).

### 2.4 Volatile compound analysis

Volatile compounds were extracted using a DVB/CAR/PDMS SPME-Arrow fiber (PAL System, Zwingen, Switzerland; phase length: 1.1 mm × 20 mm, phase thickness: 120 μm) in combination with an autosampler (PAL RSI 85, PAL System). For the meat-based broths, 0.5 mL of broth was added to a 20 mL headspace vial, along with 1 mL of a saturated NaCl solution and 10 μL of an internal standard mixture. Similarly, for the beef flavoring ingredients, 0.5 g of beef extract or beef powder was added to the headspace vial, along with 10 μL of the same internal standard mixture. The final amounts of internal standards in each sample were 0.1 μg each of 1-hexyl alcohol-*d*_13_, toluene‑*d*_8_, and 2,4,6-trimethylpyridine, and 0.02 μg each of phenyl acetate and 3-octanone. The samples were equilibrated at 500 rpm for 20 min, followed by extraction at 1000 rpm for 30 min. Equilibration and extraction were both conducted at 40 °C. The injector temperature was set to 220 °C, and desorption was carried out in splitless mode for 5 min.

GC/MS analysis was conducted using an 8890 gas chromatograph coupled with a 7000E triple quadrupole mass spectrometer (Agilent Technologies, Santa Clara, CA, USA). The volatile compounds were separated using an HP-5MS column (60 m × 0.25 mm I.D. × 0.25 μm film thickness; Agilent Technologies). Helium was used as the carrier gas at a constant flow rate of 1.0 mL/min. The oven temperature was held at 45 °C for 5 min; increased from 45 to 100 °C at a rate of 2 °C/min; increased from 100 to 153 °C at 3 °C/min; and finally ramped to 230 °C at 20 °C/min and held for 3 min. The MS transfer line and ion source temperatures were 280 and 230 °C, respectively. Electron impact ionization was conducted with an ionization energy of 70 eV. Data acquisition was performed in two segments: full-scan mode (30–550 *m*/*z*) and selected-ion monitoring (SIM) mode.

### 2.5 Identification and quantification of volatile compounds

The volatile compounds were identified by comparing their mass spectral ratios with those in the National Institute of Standards and Technology (NIST) 2020 library, using a reverse match factor (R. Match) of ≥800. The retention index (RI) was calculated based on an alkane standard mixture (C_6_–C_40_).

$$RI_{X}=100\times \left[n+\left(N-n\right)\frac{\log\left(t_{r\left(X\right)}^{\prime }\right)-\log\left(t_{r\left(n\right)}^{\prime }\right)}{\log\left(t_{r\left(N\right)}^{\prime }\right)-\log\left(t_{r\left(n\right)}^{\prime }\right)}\right]$$

where RI_X_ is the retention index of compound X; *n* is the number of carbon atoms in the smaller *n*-alkane eluted before compound X; *N* is the number of carbon atoms in the larger *n*-alkane eluted after compound X; and *t*' is the retention time of the compound.

The volatile compounds were quantified using regression equations. These equations were established based on the ratio of peak areas (analyte/internal standard). The peak area of each compound was determined by extracting the quantifier ions. The internal standards used for the quantification of each volatile compound group were as follows: phenyl acetate for acids and esters; 1-hexyl alcohol-*d*_13_ for alcohols and furans; toluene‑*d*_8_ for aldehydes, hydrocarbons, and sulfur compounds; 3-octanone for ketones; and 2,4,6-trimethylpyridine for pyrazines.

### 2.6 Method validation

The HS-SPME-Arrow-GC/MS method was validated using a modified version of the technique reported by Ye et al. (2022). First, an odorless matrix was prepared by mixing 100 mL of meat-based broth with 100 mL of methanol, followed by rotary evaporation for 2 h to remove volatile compounds. Next, stock solutions of volatile standards were prepared by accurately weighing the standard compounds and dissolving them in methanol. Internal standards were added at consistent concentrations across all standard solutions. Finally, 10 μL of each standard solution was spiked into 0.5 mL of odorless meat-based broth.

The validation criteria included linearity, LOD, LOQ, and recovery. Calibration curves were constructed for each volatile compound by plotting the mass of the standard compound on the *x*-axis and the peak area ratio of the standard compound to the internal standard on the *y*-axis. Based on these calibration curves, the concentration of each volatile compound in the sample was determined. The LOD and LOQ were calculated as 3*σ*/*S* and 10*σ*/*S*, respectively, where *σ* is the standard deviation of the *y*-intercept and *S* is the slope obtained from regression analysis (Kim et al., 2024; Nam et al., 2025). The recovery was assessed at three different mass levels for each compound, as detailed in Table S2. The recovery (%) was calculated as follows:

$$\text{Recovery}\;\left(\%\right)=\frac{\text{Measured concentration}}{\text{Spiked concentration}}\times 100.$$

### 2.7 Sensory evaluation

#### 2.7.1 Sample presentation

The broth samples were assigned three-digit random codes to maintain blind testing conditions. The presentation order was randomized using a Williams Latin-square design (Williams, 1949). A sequential monadic order was employed, whereby panelists completely evaluated each sample before proceeding to the next, with samples presented at 5-min intervals. To prevent sensory fatigue, unsalted crackers (United Biscuits, London, UK) and bottled water (ICIS 8.0, CH Beverage, Cheongju, Korea) were provided as palate cleansers between sample evaluations.

#### 2.7.2 Participants

Seventy-eight untrained consumers (12 males, 15.4%; 66 females, 84.6%; mean age: 34.5 ± 13.2 years) participated in the sensory evaluation. Participants were pre-screened to exclude individuals with food allergies or sensory impairments. The study protocol was approved by the Institutional Review Board of Sungshin Women's University (IRB No. SSWUIRB-2025-030), and written informed consent was obtained from all participants before testing.

#### 2.7.3 Evaluation procedure

Overall, odor, and taste/flavor liking were assessed using a nine-point hedonic scale ranging from 1 (dislike extremely) to 9 (like extremely), with 5 representing neither like nor dislike. The perceived odor and taste/flavor characteristics were assessed using open-ended questions with no limit on the number of sensory attributes recorded. Evaluations were conducted in individual sensory booths within a temperature and humidity-controlled laboratory under standardized fluorescent lighting. Data collection was conducted using Compusense® (version 25.0.32593, Compusense Inc., Guelph, ON, Canada) on tablet computers. Consistent testing conditions were maintained throughout the evaluation process.

### 2.8 Statistical analysis

For volatile analysis, all experiments were conducted in triplicate (*n* = 3), and the data are presented as the mean ± standard deviation. All statistical analyses were conducted using SPSS Statistics (version 29, IBM Inc., Chicago, IL, USA), with statistical significance set at *p* < 0.05 for all tests. RSM analysis was performed using Design-Expert statistical software (version 7, Stat-Ease, Minneapolis, MN, USA). Multivariate statistical analyses were conducted using MetaboAnalyst (version 6, https://www.metaboanalyst.ca/).

For the hedonic scale liking data, one-way analysis of variance (ANOVA) was conducted with “sample” as a fixed factor and “consumer” as a random factor to determine differences in the liking scores among samples. When significant differences were detected (*p* < 0.05), Duncan's multiple range test was used for post-hoc comparisons. The sensory attributes mentioned in response to the open-ended questions were analyzed by first consolidating synonymous terms. The frequency of each attribute was then calculated for each sample. Only attributes with a frequency of ≥10%, or with significantly higher observed than expected frequencies according to per-cell chi-square tests (*p* < 0.05), are reported. Correspondence analysis with confidence ellipses was used to visualize sample–attribute relationships. All sensory data were analyzed in SPSS Statistics (IBM Inc., Chicago, IL, USA) and RStudio (Posit Software, PBC, Boston, MA, USA).

To examine the integrated relationships among volatile compounds, sensory attributes, and consumer liking, hierarchical clustering and correlation analyses were performed. Only sensory attributes showing significant differences in chi-square tests (*p* < 0.05) were included. All variables were standardized using z-score normalization to account for differing units and scales. Hierarchical clustering was conducted using Euclidean distance and Ward's method. Pearson correlation coefficients were calculated to quantify relationships among the chemical, sensory, and hedonic variables. The results were visualized in a heatmap with dendrograms, where the color intensity reflects the magnitude of the standardized correlation values.

## 3 Results and discussion

### 3.1 Optimization of extraction parameters for volatile compound analysis

**Fig. 1:**
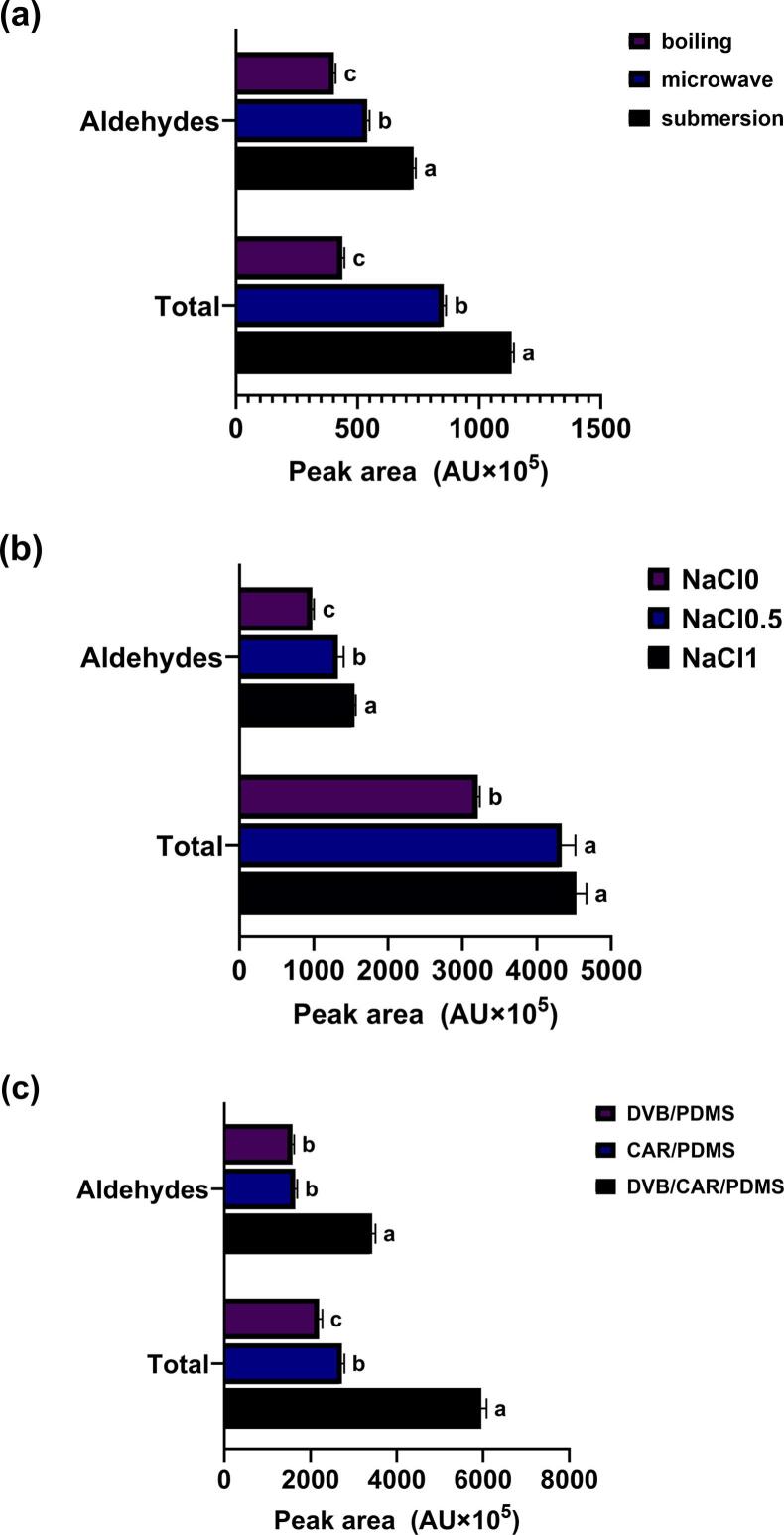
**Peak areas (AU × 10**^**5**^**) of HS-SPME-Arrow-GC/MS spectra when using different extraction conditions.** (a) Pretreatment (heating) method, (b) volume of added saturated NaCl solution, and (c) SPME-Arrow fiber type. Different lowercase letters indicate significant differences (*p* < 0.05).

The volatile compounds in meat-based broths are classified into several groups, with aldehydes being the most dominant (Guan et al., 2025; Zhang et al., 2017). Therefore, to tailor the extraction process to the matrix characteristics of meat-based broths, the extraction conditions were optimized based on the GC/MS peak areas (AU × 10^5^) of aldehydes and total volatile compounds (Fig. 1).

To determine the most effective pretreatment method for meat-based broths, techniques such as submersion, microwave heating, and boiling were used. Volatile compounds were subsequently analyzed through HS-SPME-Arrow-GC/MS, and the total peak areas (AU × 10^5^) were compared (Fig. 1a). Among the identified volatile compounds, aldehydes were the predominant group. Their total peak area was highest in samples prepared via submersion heating; therefore, this technique was established as the optimal pretreatment method. Next, the effect of NaCl addition was evaluated. Samples were prepared with 0, 0.5, and 1 mL saturated NaCl solution. The 1 mL condition yielded the highest total peak area for both aldehydes and overall volatile compounds, establishing it as the optimal concentration (Fig. 1b).

Three types of SPME-Arrow fibers (DVB/CAR/PDMS, DVB/PDMS, and CAR/PDMS) were evaluated. All fibers had a phase length of 1.1 mm × 20 mm and phase thickness of 120 μm. As shown in Fig. 1c, the DVB/CAR/PDMS SPME-Arrow fiber provided significantly higher (*p* < 0.05) GC/MS peak areas (aldehydes: 3432.45 AU × 10^5^, total volatiles: 5959.45 AU × 10^5^) than the DVB/PDMS fiber (aldehydes: 1582.24 AU × 10^5^, total volatiles: 2196.87 AU × 10^5^) and CAR/PDMS fiber (aldehydes: 1643.81 AU × 10^5^, total volatiles: 2731.83 AU × 10^5^). The selection of an appropriate coating material is critical for optimizing SPME-Arrow extraction. DVB/CAR/PDMS fibers, consisting of three distinct sorbent phases, have a high affinity for both polar and non-polar compounds, resulting in superior extraction performance (Ahamed et al., 2023). DVB/CAR/PDMS fibers have been used for the volatile analysis of a range of foods (Li, Chen, et al., 2024; Liu, Deng, et al., 2024; Liu, Gu, et al., 2024; Meng et al., 2022; Zhang et al., 2017; Zhao et al., 2024). Based on these findings, the DVB/CAR/PDMS SPME-Arrow fiber was selected for use in this study.

### 3.2 Optimization of SPME-arrow extraction conditions

The extraction conditions were optimized using RSM in combination with BBD. The equilibration time (*X*_1_), extraction time (*X*_2_), and extraction temperature (*X*_3_) were selected as the independent variables, and the total GC/MS peak area (AU × 10^5^) of aldehydes was set as the response variable (*Y*). The relationship among *X*_1_, *X*_2_, *X*_3_, and *Y* is described as follows:

$$\begin{array}{c} Y=434.71+10.28X_{1}+25.75X_{2}+18.99X_{3}\\ -15.42X_{1}X_{2}+14.82X_{1}X_{3}-6.03X_{2}X_{3}\\ -31.11X_{1}^{2}-61.26X_{2}^{2}-58.23X_{3}^{2} \end{array}.$$

**Fig. 2:**
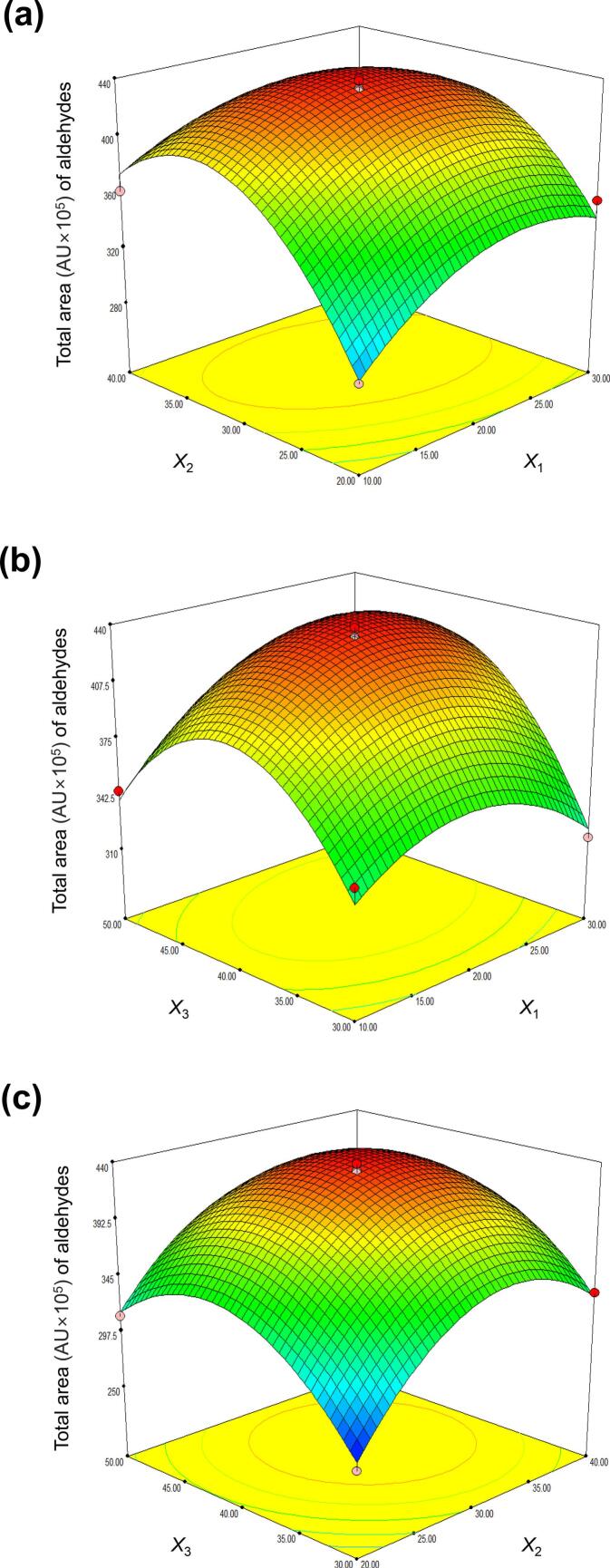
**Response surface plots for total peak area (AU × 10**^**5**^**) of aldehydes in HS-SPME-Arrow-GC/MS spectra.** The independent variables are equilibration time (*X*_1_), extraction time (*X*_2_), and extraction temperature (*X*_3_), and the response variable is the total aldehyde GC/MS peak area (*Y*). (a) *X*_1_ and *X*_2_, (b) *X*_1_ and *X*_3_, and (c) *X*_2_ and *X*_3_.

The model response was significant (F = 55.47, *p* < 0.0001), with a high coefficient of determination (*R*^2^ = 0.9862). Three-dimensional response surface plots are shown in Fig. 2. *Y* was maximized at *X*_1_ = 20 and *X*_2_ = 30 (Fig. 2a); at *X*_1_ = 20 and *X*_3_ = 40 (Fig. 2b); and at *X*_2_ = 30 and *X*_3_ = 40 (Fig. 2c). Based on these results, the optimal extraction conditions were an equilibration time of 20 min, extraction time of 30 min, and extraction temperature of 40 °C.

### 3.3 Method validation of volatile analysis

The calibration and validation results of 27 volatile compounds are presented in Table 1. The *R*^2^ values for all volatiles were greater than 0.995, indicating excellent linearity. The LODs and LOQs ranged from 0.03 to 23.59 ng and from 0.11 to 78.62 ng, respectively (LODs: 0.11–0.19 ng for alcohols, 0.03–3.31 ng for aldehydes, 3.59 ng for ethyl acetate (an ester), 0.27–5.61 ng for furans, 0.08–14.44 ng for hydrocarbons, 0.41–21.38 ng for ketones, 2.86–14.33 ng for pyrazines, and 23.59 ng for methional (a sulfur compound); LOQs: 0.36–0.65 ng for alcohols, 0.11–11.05 ng for aldehydes, 11.97 ng for ethyl acetate, 0.92–18.71 ng for furans, 0.28–48.15 ng for hydrocarbons, 1.36–71.27 ng for ketones, 9.52–47.76 ng for pyrazines, and 78.62 ng for methional). These results demonstrate that the HS-SPME-Arrow-GC/MS method is suitable and effective for the analysis of volatile compounds. The recoveries ranged from 90.69% to 109.54% (Table S2), confirming the reliability of the method. Recoveries above 100% were observed at several concentration levels; however, the magnitude of deviation was minor. For example, the recovery with the largest absolute deviation from 100% was 109.54%. This was obtained with a spiked mass of 25 ng; therefore, the absolute deviation of 9.54% corresponds to a mass of approximately 2.39 ng. Such a deviation is minor and reflects normal analytical variability related to instrumental response at low concentration levels. The RSDs ranged from 0.13% to 2.29%, demonstrating high reproducibility. Therefore, the optimized HS-SPME-Arrow-GC/MS method was suitable for analyzing volatile compounds in meat-based broths and beef flavoring ingredients.

**Table 1:** Validation parameters (calibration curves parameters, limits of detection and quantification) of the optimized HS-SPME-Arrow-GC/MS method.

Compound	Calibration curve	*R*^2^	Linear range (ng)	LOD (ng)	LOQ (ng)
***Alcohols***					
1-heptanol	*y* = 0.0163*×* − 0.0089	0.9997	0.5–40	0.11	0.36
1-octanol	*y* = 0.0063*×* − 0.0043	0.9997	1–30	0.19	0.65
***Aldehydes***					
(*E*)-2-decenal	*y* = 0.0007*×* − 0.0004	0.9997	1–20	0.28	0.93
(*E*)-2-nonenal	*y* = 0.0008*×* + 0.0001	0.9997	0.3–5	0.07	0.23
2-methylbutanal	*y* = 0.00003*×* + 0.0001	0.9997	2–75	0.47	1.58
3-methylbutanal	*y* = 0.0002*×* − 0.0003	0.9994	10–300	2.88	9.60
benzaldehyde	*y* = 0.0105*×* − 0.0234	0.9982	2–40	0.60	1.99
decanal	*y* = 0.0005*×* − 0.0002	0.9993	0.5–20	0.10	0.34
heptanal	*y* = 0.0029*×* − 0.0011	0.9999	0.5–50	0.03	0.11
hexanal	*y* = 0.0002*×* − 0.0074	0.9991	15–2000	3.31	11.05
nonanal	*y* = 0.0042*×* − 0.0022	0.9997	0.5–40	0.13	0.42
octanal	*y* = 0.0004*×* − 0.0031	0.9993	5–300	1.25	4.16
phenylacetaldehyde	*y* = 0.0001*×* − 0.0009	0.9999	10–300	1.23	4.11
***Esters***					
ethyl acetate	*y* = 0.005*×* − 0.0009	0.9999	15–250	3.59	11.97
***Furans***					
2-acetylfuran	*y* = 0.0079*×* − 0.1903	0.9990	20–500	5.61	18.71
2-furfural	*y* = 0.0013*×* − 0.0141	0.9996	20–500	4.16	13.88
2-pentylfuran	*y* = 0.0567*×* − 0.0123	0.9998	1–15	0.27	0.92
***Hydrocarbons***					
*d*-limonene	*y* = 0.0027*×* − 0.1294	0.9997	50–1000	14.44	48.15
*o*-xylene	*y* = 0.0188*×* − 0.0084	0.9989	0.5–20	0.08	0.28
*p*-xylene	*y* = 0.0173*×* − 0.0041	0.9994	0.5–40	0.15	0.50
***Ketones***					
2-butanone	*y* = 0.0005*×* − 0.0107	0.9999	100–2000	21.38	71.27
acetophenone	*y* = 0.0095*×* − 0.0058	0.9998	1.5–40	0.41	1.36
***Pyrazines***					
2,3-dimethylpyrazine	*y* = 0.0077*×* − 0.1462	0.9991	25–200	6.18	20.59
2,5-dimethylpyrazine	*y* = 0.0064*×* − 0.4127	0.9958	50–1000	14.33	47.76
methylpyrazine	*y* = 0.0042*×* − 0.0082	0.9998	10–500	2.86	9.52
trimethylpyrazine	*y* = 0.0090*×* + 0.0085	0.9997	20–1000	5.73	19.08
***Sulfur compounds***					
methional	*y* = 0.00003*×* − 0.0017	0.9994	100–500	23.59	78.62

*R*^2^, coefficient of determination; LOD, limit of detection; LOQ, limit of quantification.

### 3.4 Characterization of key volatile compounds in meat-based broths

Table 2 presents the volatile compounds analyzed using HS-SPME-Arrow-GC/MS. A total of 18 volatile compounds were identified in five meat-based broths (MB1–MB5), including 2 alcohols, 11 aldehydes, 1 ester, 1 furan, 2 hydrocarbons, and 1 ketone. A total of 13, 10, 18, 16, and 8 volatile compounds were identified in MB1, MB2, MB3, MB4, and MB5, respectively.

**Table 2:** Volatile compound concentrations (ng/mL) in five meat-based broths (MB1–MB5).

Compound	Concentration (ng/mL)					Aroma description^†^
	MB1	MB2	MB3	MB4	MB5	
***Alcohols***						
1-heptanol	69.78 ± 0.02^a^	8.92 ± 0.08^b^	2.62 ± 0.03^c^	2.89 ± 0.05^c^	nd	green, wood
1-octanol	53.73 ± 3.90^a^	5.99 ± 0.18^b^	4.99 ± 0.10^b^	6.37 ± 0.05^b^	nd	citrus, fat, fruit, green
***Aldehydes***						
(*E*)-2-decenal	13.25 ± 0.39^a^	nd	3.91 ± 0.17^c^	11.01 ± 0.17^b^	nd	fat, fish, hay, tallow
(*E*)-2-nonenal	8.68 ± 0.23^a^	nd	1.61 ± 0.13^c^	2.98 ± 0.39^b^	nd	beany, cucumber, cut grass, earth, fat
2-methylbutanal	nd	nd	93.22 ± 2.35^a^	67.36 ± 5.84^b^	nd	almond, chocolate, cocoa, fermented, hazelnut
3-methylbutanal	nd	nd	29.23 ± 1.12^a^	22.59 ± 2.29^b^	nd	acrid, almond, chocolate, cocoa, corn flakes
benzaldehyde	14.81 ± 4.02^c^	15.32 ± 0.41^b^	33.84 ± 0.06^a^	10.30 ± 0.03^e^	13.37 ± 0.14^d^	almond, berry, bitter, bitter almond, burnt sugar
decanal	24.76 ± 1.25^a^	nd	4.47 ± 0.11^b^	4.54 ± 0.10^b^	nd	fat, floral, fried, orange peel, penetrating
heptanal	91.88 ± 1.02^a^	17.42 ± 0.16^b^	7.55 ± 0.14^c^	4.00 ± 0.14^d^	7.56 ± 0.32^c^	citrus, dry fish, fat, green, nut
hexanal	3523.74 ± 113.56^a^	914.45 ± 44.33^b^	233.15 ± 1.91^c^	nd	nd	apple, cut grass, fresh, fruit, grass
nonanal	69.09 ± 1.21^a^	3.49 ± 0.22^c^	13.47 ± 0.26^b^	13.06 ± 0.11^b^	2.87 ± 0.06^c^	citrus, cucumber, fat, floral, green
octanal	519.36 ± 5.02^a^	99.25 ± 5.51^b^	57.85 ± 0.19^cd^	75.27 ± 1.31^bc^	41.31 ± 1.17^d^	citrus, fat, fruit, green, honey
phenylacetaldehyde	nd	nd	117.62 ± 5.07^a^	116.00 ± 4.60^a^	nd	berry, floral, flower, geranium, honey
***Esters***						
ethyl acetate	nd	nd	309.70 ± 3.87^b^	252.32 ± 3.15^c^	460.70 ± 24.08^a^	balsamic, fruit, grape, pineapple
***Furans***						
2-pentylfuran	23.30 ± 6.02^a^	11.30 ± 0.62^b^	6.76 ± 0.11^c^	nd	2.04 ± 0.06^d^	butter, floral, fruit, green, green bean
***Hydrocarbons***						
*o*-xylene	1.34 ± 0.02^c^	1.61 ± 0.03^c^	18.85 ± 0.18^b^	22.62 ± 0.97^a^	1.59 ± 0.02^c^	geranium
*p*-xylene	1.97 ± 0.02^c^	3.26 ± 0.04^c^	36.31 ± 0.98^b^	42.90 ± 1.80^a^	2.00 ± 0.04^c^	cold meat fat, sweet
***Ketones***						
2-butanone	nd	nd	2291.19 ± 49.12^a^	2033.68 ± 18.82^b^	nd	butterscotch, cheese, fragrant

Abbreviations: nd, not detected. ^a–e^Values in the same row with different superscript letters are significantly different (Duncan's multiple range test, *p* < 0.05). ^†^Aroma descriptions were sourced from the online database at https://www.vcf-online.nl/VcfHome.cfm.

Among the identified compounds, aldehydes were the most abundant. Benzaldehyde, heptanal, nonanal, and octanal were detected in all five meat-based broths, which is consistent with previous studies on bone soup by Meng et al. (2022) and Zhang et al. (2017). Heptanal, nonanal, and octanal were identified as key volatiles, both in this study and the previous reports, with variable importance in projection (VIP) scores exceeding 1 (Fig. S1). Notably, these aldehydes have been reported to contribute significantly to the characteristic cooked beef note (Li, Frank, et al., 2024). Aldehydes are commonly formed through lipid oxidation and degradation, as well as the Strecker degradation of amino acids (Zou et al., 2018). Owing to their typically low odor thresholds, aldehydes substantially influence the volatile profile of meat-based broths (Wang et al., 2016). Straight-chain aldehydes, including decanal, heptanal, hexanal, nonanal, and octanal, originate from the degradation of unsaturated fatty acids, such as oleic and linoleic acids. Meanwhile, branched aldehydes, such as 2-methylbutanal, 3-methylbutanal, benzaldehyde, and phenylacetaldehyde, contribute grilled meat notes (Ahamed et al., 2023).

VIP scores derived from partial least squares discriminant analysis (PLS-DA) were used to evaluate the contribution of each compound to the volatile profiles of the meat-based broths. Eleven compounds, including hexanal, 1-heptanol, 2-methylbutanal, 3-methylbutanal, 1-octanol, 2-butanone, phenylacetaldehyde, octanal, heptanal, nonanal, and decanal, had VIP scores exceeding 1, suggesting that these compounds contribute to the distinct volatile profiles of the broth samples (Fig. S1). Consequently, these compounds are suggested as candidate markers for sample differentiation.

**Fig. 3:**
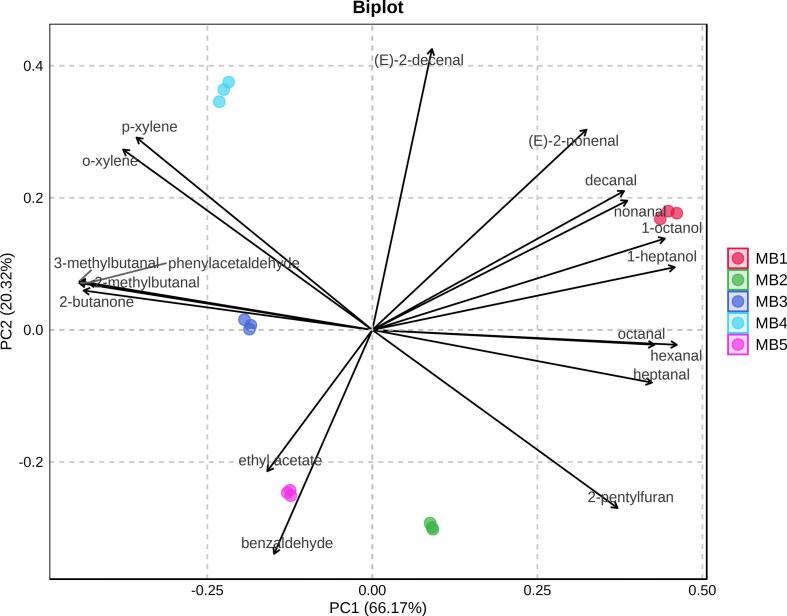
PCA biplot showing relationships between samples and volatile compounds.

Principal component analysis (PCA) was performed based on the concentrations of the 18 volatile compounds in the meat-based broths. The first and second principal components (PC1 and PC2, respectively) accounted for 66.17% and 20.32% of the total variance, respectively. Notably, the compounds with VIP > 1 contributed to the separation of samples in the PCA biplot (Fig. 3). Among them, hexanal, 1-heptanol, 1-octanol, octanal, heptanal, nonanal, and decanal were associated with the localization of MB1 samples on the positive side of PC1. MB1 had the highest detected concentration of hexanal at 3523.74 ng/mL, compared with 914.45 and 233.15 ng/mL for MB2 and MB3, respectively, and none for MB4 and MB5 (Table 2). Previous reports have identified hexanal as a predominant volatile compound in stewed beef (Zou et al., 2018), highlighting its significance in heat-treated meat products. It is primarily generated through the oxidation of polyunsaturated fatty acids, such as linoleic acid, under thermal conditions (Nam et al., 2025). MB1 also had higher concentrations of octanal (519.36 ng/mL), heptanal (91.88 ng/mL), nonanal (69.09 ng/mL), and decanal (24.76 ng/mL), which are associated with fatty notes, than other samples (*p* < 0.05). Therefore, MB1 might exhibit stronger fatty characteristics. Meanwhile, 1-heptanol (green and wood notes) and 1-octanol (citrus, fat, fruit, and green notes) have been identified in previous studies on pan-roasted beef and beef soup (Ahamed et al., 2023; Wang et al., 2022).

For MB3 and MB4, which were positioned along the negative direction of PC1 in the PCA biplot, were mainly influenced by 2-methylbutanal, 3-methylbutanal, 2-butanone, and phenylacetaldehyde. These volatile compounds were exclusively detected in MB3 and MB4 (Table 2). According to Ahamed et al. (2023), 2-methylbutanal (almond, chocolate, cocoa, fermented, and hazelnut notes) and 3-methylbutanal (acrid, almond, chocolate, cocoa, and corn flake notes) are commonly found in pan-roasted meats such as beef, pork, and chicken. Phenylacetaldehyde has been reported to impart a rosy note to broth (Wang et al., 2016). Furthermore, 2-butanone (butterscotch, cheese, and fragrant notes) has previously been documented in chicken broth (Feng et al., 2018). This compound, known for its fruity note, is also permitted as a direct food additive (Kim et al., 2021).

In conclusion, aldehydes were the predominant type of volatile compound in all five samples. However, differences in their concentrations contributed to distinct volatile profiles among the samples. The key volatile compounds identified through VIP analysis corresponded well with the clustering patterns observed in PCA, highlighting their importance in distinguishing volatile profiles.

The distinct volatile profiles of the five meat-based broth samples may be related to their ingredients (Table S1). The volatile profile of MB1 included significantly higher levels of medium- and long-chain aliphatic aldehydes (hexanal, octanal, heptanal, and nonanal) and corresponding alcohols (1-heptanol and 1-octanol) than those of the other samples. These aldehydes are known products of lipid oxidation, particularly from unsaturated fatty acids, which are abundant in beef (Li, Chen, et al., 2024). This suggests that these compounds likely originated from the beef bone extract and beef broth base in MB1. The volatile profiles of MB3 and MB4 contained Strecker aldehydes such as phenylacetaldehyde (117.62 and 116.00 ng/mL, respectively), 2-methylbutanal (93.22 and 67.36 ng/mL, respectively), and 3-methylbutanal (29.23 and 22.59 ng/mL, respectively). These compounds are formed via the Strecker degradation of phenylalanine, isoleucine, and leucine, respectively (Yang et al., 2021). The presence of amino base and vegetable extracts in these samples likely facilitated these reactions during cooking, contributing to their distinct volatile profiles. MB2 and MB5 exhibited a notably simpler volatile profile, which was likely attributed to the lack of ingredients such as amino bases and vegetable extracts. In particular, MB5 had a significantly higher concentration of ethyl acetate (460.70 ng/mL) than the other samples (*p* < 0.05). This ester has been reported to impart fruity notes to wine and pork broth (Kim et al., 2021; Wang et al., 2016). These results collectively demonstrate that the ingredient composition, especially the presence of amino acid-rich bases and fat-containing components, significantly influences the volatile profile of meat-based broth products. Variations in aldehyde content are closely related to lipid oxidation and Strecker degradation, which vary depending on the ingredient composition.

The identified volatile compounds were compared with those found in two common beef flavoring ingredients, namely, beef extract and beef powder. This comparison provides insight into similarities and differences in the volatile compounds and overall volatile profiles of meat-based products. It also offers a basis for improving the volatile composition of broths. Because ingredients specifically developed for broth are limited, beef-derived flavoring ingredients were selected as reference materials. Table S3 presents the volatile compounds in beef extract and powder, as analyzed using the same HS-SPME-Arrow-GC/MS technique. Several of the aldehydes and hydrocarbons listed in Table S3, including 3-methylbutanal, benzaldehyde, heptanal, hexanal, nonanal, phenylacetaldehyde, *o*-xylene, and *p*-xylene, were also identified in the meat-based broths (Table 2). Among these, the aldehydes 3-methylbutanal, heptanal, hexanal, nonanal, and phenylacetaldehyde were detected as key volatile compounds of the meat-based broths (VIP > 1).

2-Acetylfuran and 2-furfural were detected in high concentrations in beef powder (763.42 and 607.17 ng/g, respectively), as well as in beef extract at lower concentrations (55.69 and 66.86 ng/g, respectively), but not in the meat-based broths. These compounds are both furan derivatives, typically produced through Maillard and sugar degradation reactions. 2-Acetylfuran imparts buttery and meaty notes, as previously reported in studies on cooked meats (Sohail et al., 2022; Zhang et al., 2022). Meanwhile, 2-furfural has notes of almonds, baked potatoes, bread, burnt sugar, and candy. Its presence in beef powder and beef extract aligns with a prior study on the volatile profile of beef (Liu, Gu, et al., 2024). Four pyrazines (2,3-dimethylpyrazine, 2,5-dimethylpyrazine, methylpyrazine, and trimethylpyrazine) and one sulfur compound (methional) were found in the beef flavoring ingredients. Notably, no compounds of these types were detected in any of the commercial meat-based broths. Pyrazines are volatile nitrogen-containing heterocyclic compounds that impart roasted, baked, and nutty notes to foods (Nie et al., 2025; Yu et al., 2021). These compounds are primarily created through the Maillard reaction and greatly improve the volatile profile of meat-based products (Yu et al., 2021). Methional, a sulfur-based volatile compound, has been found in cooked beef, stewed beef, and pork broth, where it adds pleasant warm-meat, brothy, and cooked-potato notes (Liu, Deng, et al., 2024; Wang et al., 2016; Watanabe et al., 2015). Methional greatly enhances the fatty and floral notes of pork broth because it has a low odor threshold (Wang et al., 2016). The methional in the beef extract, which may have formed through thiamine degradation or the Maillard reaction, likely contributed to the meaty note (Zhao et al., 2024). In contrast, in the meat-based broths, aldehydes mainly contributed to the fatty note and sulfur compounds were absent.

The Maillard reaction is widely known for its role in forming characteristic meat notes (Chu et al., 2025; Deng et al., 2025). In thermally processed meat, compounds like aldehydes, furans, and pyrazines contribute significantly to the volatile profile (Nie et al., 2024; Min et al., 2026). Furan derivatives such as 2-acetylfuran and 2-furfural are formed through Maillard and sugar degradation reactions during the thermal processing of meat, and impart sweet, caramel, and smoky notes that improve the sensory quality of meat products (Yin et al., 2022; Zhang et al., 2022). Pyrazines like 2,5-dimethylpyrazine are produced by the interaction of sugars, amino acids, and lipids during the Maillard reaction and impart roasted, nutty, and toasted notes (Min et al., 2026), thereby enhancing the volatile profile further (Nie et al., 2024). The Maillard reaction also produces various sulfur compounds that enhance the roasted notes and umami taste of beef (Nie et al., 2024; Min et al., 2026). Therefore, the development of Maillard reaction-based flavoring agents is essential for improving the volatile profile and sensory qualities of meat-based broths.

### 3.5 Consumer acceptance and sensory profiling of meat-based broths

Consumer acceptance of the five meat-based broth samples was assessed based on overall, odor, and taste/flavor liking across a nine-point hedonic scale (1 = dislike extremely, 5 = neither like nor dislike, 9 = like extremely). Significant differences were observed among samples for all sensory parameters (*p* < 0.001, Table 3). MB3 demonstrated the highest scores for overall liking (6.00), odor (6.23), and taste/flavor (5.99). In contrast, MB2 exhibited the lowest scores, with 4.97 for overall liking, 4.79 for odor, and 4.60 for taste/flavor. MB1, MB4, and MB5 received intermediate acceptance, with scores clustered around the neutral rating of 5 (neither like nor dislike).

**Table 3:** Consumer liking scores for five meat-based broth samples (MB1–MB5).a–c

Liking	Samples				
	MB1	MB2	MB3	MB4	MB5
Overall	5.87 ± 1.61^ab^	4.97 ± 1.81^c^	6.00 ± 1.34^a^	5.35 ± 1.62^bc^	5.62 ± 1.63^ab^
Odor	5.49 ± 1.53^b^	4.79 ± 1.57^c^	6.23 ± 1.54^a^	5.63 ± 1.52^b^	5.40 ± 1.37^b^
Taste/Flavor	5.77 ± 1.67^ab^	4.60 ± 1.94^d^	5.99 ± 1.57^a^	5.17 ± 1.72^c^	5.38 ± 1.75^bc^

a–c Values in the same row with different superscript letters are significantly different (Duncan's multiple range test, *p* < 0.001).

Correspondence analysis was conducted to examine the relationships between samples and sensory attributes based on frequency data (Table S4, Fig. S2). The first two dimensions explained 52.12% and 26.45% of the total variance, respectively, with a cumulative variance of 78.57%.

MB3, positioned on the positive axis of Dim 1, was strongly associated with strong, meat, meat broth, and soy sauce odor attributes, as well as meat broth flavor. Conversely, MB2, located on the negative axis of Dim 1, was characterized by weak odor and weak salty taste attributes, together with weak, milk, and clean flavor attributes. Dim 1 therefore represents sensory differentiation among samples based on odor and flavor intensity. Notably, this differentiation showed a strong association with consumer acceptance. MB3, characterized by intense meaty odor and flavor and pronounced umami notes, exhibited the highest consumer liking scores, whereas MB2, perceived as weak in odor and flavor and insufficiently salty, had the lowest liking scores. These observations align with previous findings on sensory perceptions of broths. Kwon et al. (2011) demonstrated that beef stock samples with strong beef odor and flavor achieved superior consumer liking scores, and Jung et al. (2010) reported that consumers favored samples with higher perceived intensities of beef, seasoning, and MSG flavors. Similarly, Li et al. (2022) established positive correlations between meat and seasoning odor attributes and odor liking, as well as between perceived saltiness and taste liking in bone broth products.

MB1, MB4, and MB5, located at the center of Dim 1, displayed moderate flavor intensities that corresponded to their intermediate liking scores. Among these, MB4 had distinctive MSG odor and seasoning flavor notes (Table S4) and received slightly lower liking scores than MB1 and MB5. Although MSG and seasoning generally enhance consumer liking, excessive intensities may reduce acceptance. This finding aligns with those of Essed et al. (2009), who demonstrated that consumer liking decreased when MSG concentrations exceeded optimal levels.

### 3.6 Correlation analysis among volatile compounds, sensory attributes, and consumer liking

Fig. S3 presents a heatmap illustrating the relationships among volatile compounds, sensory attributes, and consumer liking across the samples. 2-Methylbutanal, 3-methylbutanal, and phenylacetaldehyde showed strong positive correlations with meat odor and were particularly prominent in MB3 and MB4. Notably, benzaldehyde was closely associated with meat broth odor and flavor, contributing to the pronounced meaty aroma and broth flavor notes of MB3. The abundance of these compounds aligned well with MB3's high consumer liking scores. In contrast, MB2 and MB5 displayed relatively simple volatile profiles and showed negative correlations with flavor intensity, which was reflected in their low overall acceptance.

## 4 Conclusion

This study confirms that the optimized HS-SPME-Arrow-GC/MS method can reliably characterize the volatile profiles of meat-based broths. Under the optimized conditions, the method achieved low LODs and LOQs and acceptable recovery, demonstrating adequate sensitivity and accuracy for detecting and quantifying volatile compounds in meat-based broths.

Eighteen volatile compounds were identified in five commercial meat-based broths, with aldehydes associated with fatty notes being the primary contributors to the volatile profile. MB1 had higher concentrations of fat-associated aldehydes and alcohols, whereas MB3 and MB4 exhibited more roasted or broth-like notes. A comparison with beef flavoring ingredients emphasized the potential for enhancing the volatile profile of meat-based broths. Notably, pyrazines and methional, which contribute roasted and cooked characteristics, were found exclusively in the beef flavoring ingredients. These compounds are formed through the Maillard reaction, suggesting that using seasonings developed via this reaction could enhance the volatile profiles of meat broths. Furthermore, sensory evaluation supported these findings, as broths with a strong meaty aroma, rich umami flavor, and balanced saltiness received significantly higher overall liking scores. Overall, these findings provide valuable insights for improving the volatile profiles of Korean-style HMR products, particularly meat-based broths.

In the future, ingredients or seasonings should be developed based on the key volatile compounds identified in this study and added to meat-based broths to investigate whether they offer improved sensory profiles. Research in this area is expected to aid the development of more appealing meat-based broths that better align with consumer preferences.

## Glossary

**Table:** 

HS-SPME-Arrow-GC/MS	headspace solid-phase microextraction-Arrow combined with gas chromatography/mass spectrometry
RSM	response surface methodology
BBD	Box–Behnken design

## CRediT authorship contribution statement

**Jin-Kyung Nam:** Writing – original draft, Methodology, Investigation, Data curation. **Mi-Ran Kim:** Writing – original draft, Data curation. **Jeong Eun Hyeon:** Resources, Project administration, Conceptualization. **Hae Won Jang:** Writing – review & editing, Supervision, Resources, Project administration, Conceptualization.

## Funding

This work was supported by Korea Institute of Planning and Evaluation for Technology in Food, Agriculture, and Forestry (IPET) through High Value-added Food Technology Development Program, funded by 10.13039/501100003624Ministry of Agriculture, Food and Rural Affairs (MAFRA) [grant number: RS-2024-00407170].

## Declaration of competing interest

The authors declare that they have no known competing financial interests or personal relationships that could have appeared to influence the work reported in this paper.

## Data Availability

Data will be made available on request.

## Appendix A Supplementary data

Supplementary material Image 1
