# Supplementary material for: Characterization of key volatile compounds in meat-based broths using HS-SPME-Arrow-GC/MS and their relationship to sensory perception
Source: Food Chem X. 2026 Jan 27;34:103601. doi: 10.1016/j.fochx.2026.103601 (PMC12887422; doi:10.1016/j.fochx.2026.103601)
Supplement: Supplementary file 1 — Supplementary material [file mmc1.docx]

**Supplementary Material**

**Characterization of key volatile compounds in meat-based broths using HS-SPME-Arrow-GC/MS and their relationship to sensory perception**

**Jin-Kyung Nam^a^, Mi-Ran Kim^b^, Jeong Eun Hyeon^a^, and Hae Won Jang^a,^***

^a^*Department of Food Science and Biotechnology, Sungshin Women’s University, Seoul 01133, Republic of Korea*

^b^*Department of Food Science and Nutrition, The Catholic University of Korea, Bucheon 14662, Republic of Korea*

***Corresponding author:**

*E-mail*: hwjang@sungshin.ac.kr (H. W. Jang)


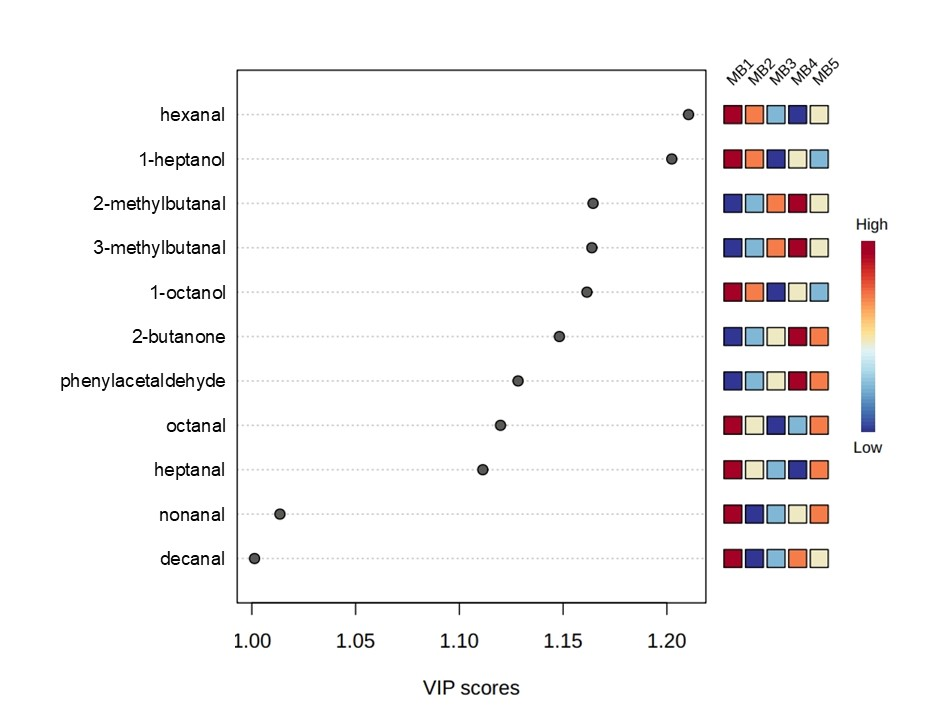


**Figure S1. Key volatile compounds in meat-based broths.**


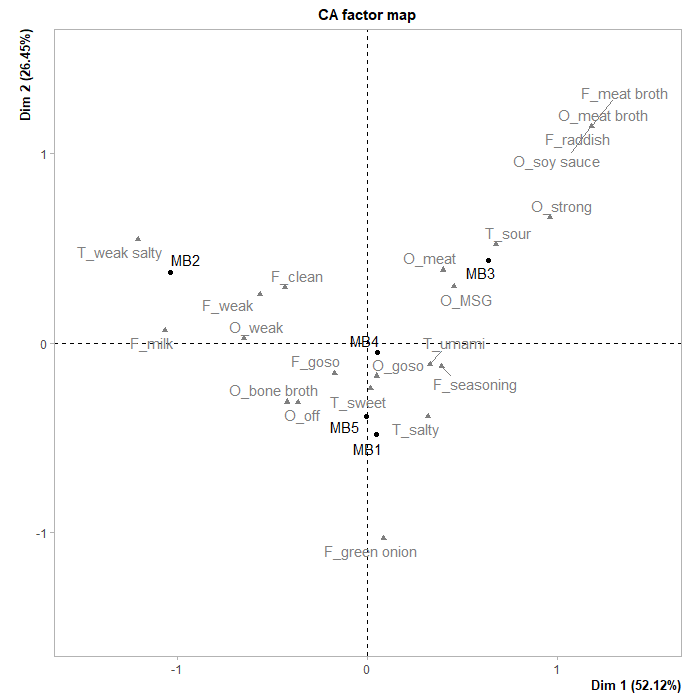


**Figure S2. CA plot of relationship between samples and sensory attributes.** Dots represent samples and triangles indicate sensory attributes. O, odor attribute; T, taste attribute, and F, flavor attribute.


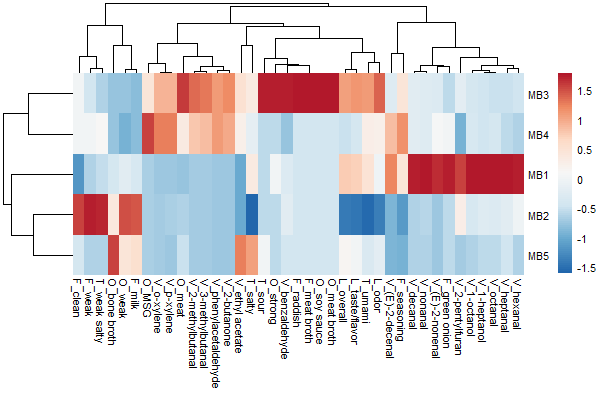


**Figure S3. Heatmap of volatile compounds, sensory attributes, and consumer liking scores for samples.** The color scale represents standardized values ranging from −1.5 (blue) to 1.5 (red). Hierarchical clustering was performed using Euclidean distance and Ward’s method. Abbreviations: V, volatile compound; L, liking score; O, odor attribute; T, taste attribute, and F, flavor attribute.

**Table S1.** Ingredient composition of commercial meat-based broth samples

| Sample | Ingredients |
| --- | --- |
| MB1 | Purified water, beef bone extract, garlic extract, beef broth base, black pepper |
| MB2 | Purified water, beef bone extract |
| MB3 | Purified water, fatty brisket slices, brisket extract, beef broth powder, radish extract powder, flavor enhancer, amino base, mixed vegetable base, complex seasoning mixture |
| MB4 | Purified water, beef bone concentrate, amino base, beef extract powder, green onion extract, flavor enhancer, liquid seasoning sauce, refined salt, mixed vegetable base, garlic extract concentrate |
| MB5 | Purified water, beef bone extract, flavor enhancer, refined salt |

**Table S2.** Recovery (%) of volatile compounds using the optimized HS-SPME-Arrow-GC/MS method

| Compound | Spiking 1 | | | Spiking 2 | | | Spiking 3 | | |
| --- | --- | --- | --- | --- | --- | --- | --- | --- | --- |
|  | Mass (ng) | Recovery (%) | RSD (%) | Mass (ng) | Recovery (%) | RSD (%) | Mass (ng) | Recovery (%) | RSD (%) |
| ***Alcohols*** |  |  |  |  |  |  |  |  |  |
| 1-heptanol | 1.5 | 98.90 ± 0.31 | 0.31 | 5 | 99.90 ± 0.64 | 0.64 | 35 | 103.62 ± 0.52 | 0.50 |
| 1-octanol | 2.5 | 103.84 ± 0.69 | 0.67 | 10 | 100.97 ± 0.96 | 0.95 | 25 | 97.94 ± 0.67 | 0.69 |
| ***Aldehydes*** |  |  |  |  |  |  |  |  |  |
| (*E*)-2-decenal | 2.5 | 109.29 ± 0.33 | 0.30 | 5 | 102.87 ± 0.69 | 0.67 | 7.5 | 94.70 ± 0.32 | 0.34 |
| (*E*)-2-nonenal | 1 | 92.29 ± 0.50 | 0.54 | 2 | 95.52 ± 0.70 | 0.73 | 4 | 99.26 ± 0.69 | 0.70 |
| 2-methylbutanal | 15 | 101.42 ± 0.57 | 0.56 | 30 | 97.50 ± 0.32 | 0.33 | 45 | 95.42 ± 0.54 | 0.56 |
| 3-methylbutanal | 15 | 104.39 ± 0.42 | 0.40 | 50 | 100.44 ± 0.70 | 0.70 | 100 | 93.70 ± 0.31 | 0.33 |
| benzaldehyde | 5 | 108.96 ± 0.68 | 0.63 | 10 | 101.99 ± 0.34 | 0.33 | 20 | 93.82 ± 0.26 | 0.28 |
| decanal | 2.5 | 93.86 ± 0.35 | 0.37 | 5 | 101.69 ± 0.67 | 0.65 | 10 | 109.54 ± 0.58 | 0.53 |
| heptanal | 5 | 101.01 ± 0.16 | 0.16 | 10 | 97.97 ± 0.26 | 1.29 | 45 | 92.36 ± 0.34 | 0.36 |
| hexanal | 100 | 108.96 ± 0.65 | 0.60 | 500 | 103.42 ± 0.99 | 0.95 | 1500 | 98.56 ± 0.32 | 1.34 |
| nonanal | 2.5 | 106.77 ± 0.62 | 0.58 | 5 | 102.67 ± 0.61 | 0.59 | 35 | 99.64 ± 0.64 | 0.64 |
| octanal | 25 | 107.56 ± 0.72 | 0.67 | 50 | 104.92 ± 0.61 | 0.58 | 250 | 101.63 ± 0.66 | 0.65 |
| phenylacetaldehyde | 50 | 109.50 ± 0.54 | 0.49 | 100 | 102.44 ± 0.61 | 0.59 | 250 | 101.11 ± 0.52 | 0.51 |
| ***Esters*** |  |  |  |  |  |  |  |  |  |
| ethyl acetate | 100 | 98.05 ± 0.62 | 0.64 | 150 | 102.54 ± 0.25 | 0.24 | 200 | 105.95 ± 0.15 | 0.14 |
| ***Furans*** |  |  |  |  |  |  |  |  |  |
| 2-acetylfuran | 25 | 109.54 ± 0.15 | 0.14 | 200 | 98.14 ± 0.26 | 0.26 | 400 | 93.16 ± 0.97 | 1.04 |
| 2-furfural | 30 | 96.45 ± 0.38 | 0.39 | 150 | 102.05 ± 0.13 | 0.13 | 300 | 105.54 ± 0.08 | 1.03 |
| 2-pentylfuran | 2.5 | 102.55 ± 0.76 | 0.74 | 5 | 96.72 ± 0.29 | 0.30 | 10 | 90.69 ± 0.74 | 0.81 |
| ***Hydrocarbons*** |  |  |  |  |  |  |  |  |  |
| *d*-limonene | 250 | 101.64 ± 0.42 | 0.42 | 500 | 98.61 ± 0.18 | 0.19 | 750 | 94.54 ± 0.80 | 0.85 |
| *o*-xylene | 2.5 | 106.22 ± 0.32 | 0.30 | 5 | 100.20 ± 2.29 | 2.29 | 10 | 96.17 ± 0.16 | 0.16 |
| *p*-xylene | 2.5 | 103.73 ± 0.47 | 0.45 | 10 | 99.59 ± 0.02 | 1.02 | 20 | 99.15 ± 0.37 | 0.37 |
| ***Ketones*** |  |  |  |  |  |  |  |  |  |
| 2-butanone | 500 | 96.31 ± 0.26 | 0.27 | 1000 | 100.01 ± 0.90 | 0.90 | 1500 | 106.25 ± 0.66 | 0.62 |
| acetophenone | 2.5 | 94.48 ± 0.48 | 0.51 | 10 | 96.24 ± 0.51 | 0.53 | 25 | 99.06 ± 0.26 | 0.26 |
| ***Pyrazines*** |  |  |  |  |  |  |  |  |  |
| 2,3-dimethylpyrazine | 50 | 106.48 ± 0.80 | 0.75 | 75 | 100.82 ± 0.58 | 0.58 | 100 | 96.59 ± 0.41 | 0.42 |
| 2,5-dimethylpyrazine | 250 | 105.02 ± 0.63 | 0.60 | 500 | 99.85 ± 0.49 | 0.49 | 750 | 93.01 ± 0.44 | 0.47 |
| methylpyrazine | 75 | 105.43 ± 0.44 | 0.41 | 150 | 99.36 ± 0.40 | 1.41 | 250 | 95.04 ± 0.75 | 0.79 |
| trimethylpyrazine | 40 | 101.33 ± 0.31 | 0.31 | 200 | 99.16 ± 0.78 | 0.79 | 400 | 99.00 ± 0.80 | 0.81 |
| ***Sulfur compounds*** |  |  |  |  |  |  |  |  |  |
| methional | 150 | 93.98 ± 0.91 | 0.97 | 250 | 101.85 ± 0.92 | 0.91 | 300 | 107.00 ± 0.97 | 0.91 |

**Table S3.** Volatile profiles (ng/g) of beef extract and powder

| Compound | Concentration (ng/g) | |  | *p* value | Aroma description^†^ |
| --- | --- | --- | --- | --- | --- |
|  | Beef extract | Beef powder | |  |  |
| ***Aldehydes*** |  |  | |  |  |
| 3-methylbutanal | 105.92 ± 3.81^b^ | 192.14 ± 1.34^a^ | | ** | acrid, almond, chocolate, cocoa, corn flakes |
| benzaldehyde | 7.45 ± 0.04^b^ | 41.38 ± 2.23^a^ | | ** | almond, berry, bitter, bitter almond, burnt sugar |
| heptanal | nd | 9.98 ± 0.55^a^ | | ** | citrus, dry fish, fat, green, nut |
| hexanal | nd | 159.69 ± 1.24^a^ | | ** | apple, cut grass, fresh, fruit, grass |
| nonanal | 1.13 ± 0.01^b^ | 8.23 ± 0.30^a^ | | ** | citrus, cucumber, fat, floral, green |
| phenylacetaldehyde | 192.83 ± 15.56^b^ | 541.33 ± 18.70^a^ | | * | berry, floral, flower, geranium, honey |
| ***Furans*** |  |  | |  |  |
| 2-acetylfuran | 55.69 ± 0.31^b^ | 763.42 ± 26.91^a^ | | ** | balsamic, cocoa, coffee, fermented |
| 2-furfural | 66.86 ± 3.77^b^ | 607.17 ± 3.47^a^ | | ** | almond, baked potatoes, bread, burnt sugar, candy |
| ***Hydrocarbons*** |  |  | |  |  |
| *d*-limonene | nd | 1026.93 ± 10.53^a^ | | ** | citrus, lemon, mint |
| *o*-xylene | 1.13 ± 0.01^b^ | 4.16 ± 0.05^a^ | | ** | geranium |
| *p*-xylene | 1.24 ± 0.03^b^ | 9.83 ± 0.18^a^ | | ** | cold meat fat, sweet |
| ***Ketones*** |  |  | |  |  |
| acetophenone | 3.39 ± 0.13^b^ | 51.11 ± 0.81^a^ | | * | almond, animal, floral, flower |
| ***Pyrazines*** |  |  | |  |  |
| 2,3-dimethylpyrazine | nd | 132.20 ± 1.65^a^ | | ** | caramel, cocoa, coffee, dry, hazelnut |
| 2,5-dimethylpyrazine | nd | 1118.23 ± 12.16^a^ | | ** | burnt, cocoa, coffee |
| methylpyrazine | 488.95 ± 39.87^a^ | 177.34 ± 3.10^b^ | | ** | burnt, cocoa, fish, green |
| trimethylpyrazine | 83.90 ± 4.66^b^ | 767.64 ± 18.15^a^ | | ** | bread, burnt, cocoa, coffee, earth |
| ***Sulfur compounds*** |  |  | |  |  |
| methional | 327.44 ± 8.57^a^ | nd | | ** | baked potatoes, caramel, cooked potato, earth, meat |

Abbreviations: nd, not detected. ^a,b^Values in the same row with different superscript letters are significantly different (Student’s *t*-test, **p* < 0.01, ***p* < 0.001). ^†^Aroma descriptions were sourced from the online database at https://www.vcf-online.nl/VcfHome.cfm.

**Table S4.** Frequency of sensory descriptors described by consumers for five meat-based broths (MB1–MB5)

| Descriptors | Sample | | | | |
| --- | --- | --- | --- | --- | --- |
|  | MB1 | MB2 | MB3 | MB4 | MB5 |
| ***Odor attributes*** |  |  |  |  |  |
| O_weak | 7 | 17^(+)***^ | 4^(−)***^ | 3 | 11 |
| O_strong | 1 | 0 | 4^(+)***^ | 0 | 0 |
| O_meat | 3^(−)*^ | 4 | 20^(+)***^ | 11 | 5 |
| O_meat broth | 0 | 0 | 8^(+)***^ | 0 | 0 |
| O_bone broth | 2 | 4 | 1 | 1 | 7^(+)***^ |
| O_soy sauce | 0 | 0 | 4^(+)***^ | 0 | 0 |
| O_MSG | 0 | 0 | 4 | 8^(+)***^ | 0 |
| O_*goso* | 14 | 7 | 12 | 10 | 11 |
| O_off | 8 | 6 | 2 | 2 | 5 |
| ***Taste attributes*** |  |  |  |  |  |
| T_sweet | 9 | 4 | 6 | 5 | 6 |
| T_sour | 1 | 1 | 13^(+)***^ | 1 | 4^(+)***^ |
| T_salty | 30 | 0^(−)***^ | 30 | 22 | 41 |
| T_weak salty | 2^(−)*^ | 23^(+)***^ | 1^(−)***^ | 8 | 1^(−)***^ |
| T_umami | 12 | 2^(−)***^ | 15 | 11 | 8 |
| ***Flavor attributes*** |  |  |  |  |  |
| F_weak | 5 | 16^(+)***^ | 6 | 8 | 5 |
| F_meat broth | 0 | 0 | 6^(+)***^ | 0 | 0 |
| F_raddish | 0 | 0 | 3^(+)***^ | 0 | 0 |
| F_green onion | 4^(+)*^ | 0 | 0 | 1 | 0 |
| F_milk | 1 | 5^(+)***^ | 0 | 0 | 3 |
| F_seasoning | 7 | 0^(−)***^ | 7 | 10^(+)*^ | 1 |
| F_*goso* | 11 | 8 | 6 | 8 | 6 |
| F_clean | 2 | 9^(+)***^ | 5 | 5 | 4 |

Abbreviations: O, odor attribute; T, taste attribute, and F, flavor attribute. Per-cell chi-square test results are reported for each cell, with (+) and (−) indicating frequencies higher or lower than expected, respectively (^*^*p* < 0.05, ^**^*p* < 0.01, ^***^*p* < 0.001).
